# Sustainable practices and ophthalmological services: a caterpillar effect?

**Published:** 2020

**Authors:** Mădălina Gheorghe Consuela

**Affiliations:** * Philologist, Authorized translator

Nowadays, the concept “sustainability” has many meanings for different people as well as for different fields. Not until recently specialists have debated the appropriate measures to develop sustainable practices in health care services as well. Usually, in the health care services, sustainability implies a reconciliation of the environmental, social, and economic demands that determine the resource usage, so as to enable “sustainable development”— the resource model allocation that allows the delivery of actual and potential health care needs, whereas maintaining a low influence on the environment, with diminished impact on the satisfaction of the needs of future generations (United Nations, 1987). In addition, health care professionals should struggle to come up with strategies to address the changes that take place continuously at the social, environmental and economic levels, and, at the same time, try to maximize the health care consumers’ safety. 

In other words, according to the article “Sustainability of public health interventions: where are the gaps?” written by Walugembe DR et al., sustainability refers to “the maintenance of health benefits over time”. 

Moreover, the author of the book “Eye Care in Developing Nations. Fourth Edition”, Larry Schwab, affirmed that some health care specialists define sustainability as having a grant to cover the ongoing costs for the next 5 years, covering costs entirely from health care consumer revenue, or combining income sources to support medical services. However, health care services will not be sustainable if quality is not sustainable. At the same time, increasing the volume of health care consumers while maintaining quality is critical. 

Moreover, according to Lennox L et al., sustainable practices become efficient if their outcomes in terms of health benefits, activities or workforce capacity are maintained. The concept of sustainability as a “process” rather than an “outcome” refers to adaptation, learning and continuous development. 

Consequently, sustainability has gained a lot of ground and attention from the specialists in the Ophthalmology field in the past few years; the possible reason being that it represents a key strategy in creating an eye care programme, through which ophthalmological services are being delivered to health care consumers. The concept of sustainable ophthalmological services may also refer to the carbon footprint reduction strategies. 

The fundamental sustainable oriented directions in ophthalmological services are: leadership, core services and management, financial viability and community (http://www.v2020eresource.org/home/newsletter/news102011). 

Nevertheless, the process of implementing sustainable ophthalmological services starts with the needs of the health care consumers and the medical staff, but, at the same time, reducing the costs, improving health care consumer care and the health care organization credentials by elaborating and implementing social responsibility strategies. 

As far as reducing costs is concerned, the provision of sustainability in health care services may lead to costs recovery, Lennox L et al. state in their paper “Navigating the sustainability landscape: a systematic review of sustainability approaches in healthcare”. Recovering costs represents the ability of an organization to produce outputs of enough value so that it acquires enough inputs to continue the production and delivery at a steady or growing rate. Further, improving health care consumer care should be the main priority of the health care organizations and prevention is the best sustainable key action. 

Last but not least, Walugembe DR et al. concluded that the main reasons for adopting sustainable strategies should become a priority for specialists in ophthalmological services because the sustained programmes’ long-term effects are easier to investigate since they are often maintained over a longer period of time; health care changes in a community become visible after 3-10 years after the beginning of the sustainable programmes; a focus on sustainability needs the consideration of the potential loss of investments for organizations and people, and the discontinuation of interventions without sustained benefits of any kind; discontinuation of interventions without planning or intention triggers a disillusionment effect for the participants and raises obstacles for future community mobilization efforts. 

**Assist. Prof. Gheorghe Consuela-Mădălina, PhD,** **Philologist, Authorized translator**

